# The Experience of COVID-19 Visitor Restrictions among Families of People Living in Long-Term Residential Care Facilities during the First Wave of the Pandemic in Ireland

**DOI:** 10.3390/ijerph19116559

**Published:** 2022-05-27

**Authors:** Nicola Cornally, Caroline Kilty, Catherine Buckley, Rónán O’Caoimh, Mark R. O’Donovan, Margaret P. Monahan, Caroline Dalton O’Connor, Serena Fitzgerald, Irene Hartigan

**Affiliations:** 1 Catherine McAuley School of Nursing and Midwifery, University College Cork, T12AK54 Cork, Ireland; caroline.kilty@ucc.ie (C.K.); megmonahan98@icloud.com (M.P.M.); c.doconnor@ucc.ie (C.D.O.); serena.fitzgerald@ucc.ie (S.F.); i.hartigan@ucc.ie (I.H.); 2 Northridge House Education and Research Centre, St. Luke’s Home, T12H970 Cork, Ireland; catherine.buckley@stlukeshome.ie; 3 Department of Geriatric Medicine, Mercy University Hospital, T12WE28 Cork, Ireland; ronan.ocaoimh@ucc.ie; 4 Health Research Board Clinical Research Facility, University College Cork, Mercy University Hospital, T12WE28 Cork, Ireland; markodonovan@ucc.ie

**Keywords:** COVID-19, long-term residential care, nursing home, family caregivers, qualitative, thematic analysis

## Abstract

Public health responses to COVID-19 in long-term residential care facilities (LTRCFs) have restricted family engagement with residents. These restrictions impact on quality of care and the psychosocial and emotional well-being of family caregivers. Following a national cross-sectional web-based survey, respondents were invited to provide personal reflections on visitor restrictions. This study aims to describe the consequences of these restrictions for individuals living in LTRCF and their families during the first wave of the COVID-19 pandemic. Data from open-ended questions contained within the survey were analyzed using Braun and Clarke’s (2006) method of thematic analysis. Four themes were identified: 1. Altered Communication and Connection; 2. Emotional and Psychological Impact; 3. Protecting and Caring Role of Staff; 4. Family Role. Throughout the narrative accounts, it is evident that the visitor restrictions impacted on the emotional and mental well-being of families. Some respondents expressed frustration that they could not assist staff in essential care provision, reducing meaning and purpose in their own lives. COVID-19 LTRCF visitor restrictions made little distinction between those providing essential personal care and those who visit for social reasons. A partnership approach to care provision is important and should encompass strategies to maintain the psychosocial and emotional well-being of families and their relatives during times of self-isolating or restrictive measures.

## 1. Introduction

The World Health Organization issued public health guidance in 2020 that resulted in public health and social measures and large-scale movement restrictions internationally [1]. Public health actions in response to COVID-19 in long-term residential care facilities (LTRCF) resulted in the cocooning of residents, restricting family engagement in caregiving and visitation. People living in congregated settings have been severely impacted by COVID-19. Older adults, people living with frailty [2] and chronic medical conditions are at risk for poorer outcomes from COVID-19 [3]. Visitor restrictions resulted in the near exclusion of family involvement in their relative’s care, potentially having a significant impact on the quality and safety of their care. Families who are central to care delivery have been required to rapidly change their approach and engagement with staff to support and connect with residents.

This is important because family involvement is pivotal in the ongoing wellbeing and resilience of residents as they act as advocates, communicators and allies [4]. Owing to their levels of insight and commitment, family members can continue to enhance the lives of persons in LTRCF [5]. Families provide socioemotional support and assist with personal care [6]. They often assist with instrumental activities of daily living, for example washing clothes, buying items, and attending external appointments with the person [7,8], in addition to assisting with decision making and care planning [7]. Family members can observe changes in the resident’s physical health before nurses do [9], and keep staff informed [8]. In some instances, relatives also help with care, nutrition, cleansing and dressing during the last few months of life [10].

Continuing to engage in care is important for families, who want to spend time with their relatives in order to maintain their relationships [7]. Family members have reported experiencing gratitude for the ability to continue to provide care in LTRCFs [5]. However, COVID-19 is impacting negatively on family-resident centered care. When visitation is restricted, it disrupts bonds and coping mechanisms between family and residents [11]. COVID-19 visitor restrictions have impacted the mental health and well-being of older persons [12], potentially leading to a greater risk of death from social isolation and loneliness [13,14].

The exclusion of families from their relatives’ care during COVID-19 has potentially impacted quality and safety—families who were integral to care provision have had to adapt to remote engagement and are now often regarded as ‘visitors’ with limited access. If unable to attend to caring responsibilities, families may experience negative emotions and altered connections with the resident and the care team [5]. Evidence suggests that some families find it challenging to give or receive information from staff under the current restrictions [15]. In some cases, staff have facilitated calls/video messages between families and residents to reduce the distress and loneliness felt from not being able to be present in person [16,17].

While there is emerging evidence that the restrictions have impacted negatively on the psychological and emotional well-being of families [15], there is limited in depth data on their experience of these restrictions [18,19]. Hence, this paper presents the reflections from 120 families during the ‘harshest’ visitor restrictions to LTRCF in Ireland during the first wave of COVID-19.

## 2. Materials and Methods

### 2.1. Design

The first part of the Engaging Remotely in Care (ERiC) study, included a cross-sectional, web-based survey, conducted nationally to investigate the psychosocial impact of COVID-19 visitor restrictions on family and friends of people residing in LTRCF using a variety of tools and measures [15]. As part of this initial phase, respondents were also invited to provide personal reflections and accounts of their experiences with visiting restrictions and changes in their involvement in care during the first 3 months of COVID-19 in Ireland (from March 2020) via open-ended questions contained within the survey. The aim of this qualitative element was to describe the consequences of the visitor restrictions during the COVID-19 pandemic for individuals living in LTRCF and their families from the perspective of those impacted by the public health measures. Three open-ended questions were used to capture this information:

1. What are your concerns regarding your relatives/friends care during the COVID-19 outbreak?
2. Please specify the areas that you need information about with reference to COVID-19 in Residential Care Facilities
3. If you have time to reflect, please share your personal experiences of how the restrictive public health measures have impacted on you and your involvement in your relative’s/friends’ care

This paper focuses exclusively on the qualitative findings from the open-ended questions contained in the national survey. 

### 2.2. Sample & Recruitment

Data were collected using convenience sampling for two weeks up until 30 June 2020. The survey link was circulated on social media accounts of local and regional newspapers, University emailing lists, the Nursing Homes Ireland webpage and through professional networks of the research team. Family, friends, and legal guardians of residents living in LTRCFs in Ireland were eligible to participate. The survey tool was piloted with family members (n = 4) prior to distribution Respondents were informed about the nature and purpose of the study, in addition to any risks and benefits prior to consenting. All data collected and analyzed were anonymous. Ethical approval was granted by the Social Research Ethics Committee at University College Cork, Ireland. Detailed descriptive statistics of the sample included in this analysis are available in Supplementary Table S1. In summary 120 respondents completed the open-ended question reflecting on their personal experiences and were included in this analysis. Over half (69%) were aged between 45–64 years and most were frequent visitors to nursing homes with almost 81% visiting at least once a week. Approximately 83% (n = 99) of the respondents were female. Most, 95% (n = 114) were a family member of the resident, 24% (n = 29) lived alone and 64% (n = 77) respondents were employed. Of 109 participants answering the applicable questions, 70% (76/109) of the residents were reported to have dementia and an additional 12% (13/109) were reported to have cognitive impairment. The majority of respondents represented residents living in LTRCFs situated in an urban areas (n = 51, 42.5%). A smaller percentage were in rural (n = 33, 27.5%) and suburban (n = 36, 30.0%) areas. 

### 2.3. Data Analysis

Demographic and other quantitative data were analyzed in SPSS V24 using descriptive statistics (frequencies and percentages). The rich textual data were analyzed using reflexive Thematic Analysis. This is a pragmatic qualitative method of data analysis [20]. The reflexive component offers the qualitative researcher a degree of flexibility in relation to how thematic analysis is enacted, whilst also providing a guiding framework [21]. It calls for reflective and thoughtful engagement with data and with the analytic process [21] and allows for analysis of both semantic and latent data [22], at levels of description and interpretation [23]. The phases of Thematic Analysis [20] (p. 87) are (1) process of familiarization with the data, (2) generating initial codes, (3) searching for themes, (4) reviewing themes, (5) defining and naming themes and (6) producing the report. However, it is suggested that a recursive rather than linear process is followed, insofar as the researcher has scope to move back and forth between steps [20]. The process involved study authors (CK, MM, NC, IH) reviewing respondent accounts to identify salient features within the data and assigning preliminary codes to these. Codes were then reviewed to organize all relevant data and collated them into potential themes. These potential themes were again reviewed amongst the research team, and cross checked with the dataset, before further refinement and the construction of final illustrative and coherent themes.

## 3. Results

**Figure 1:**
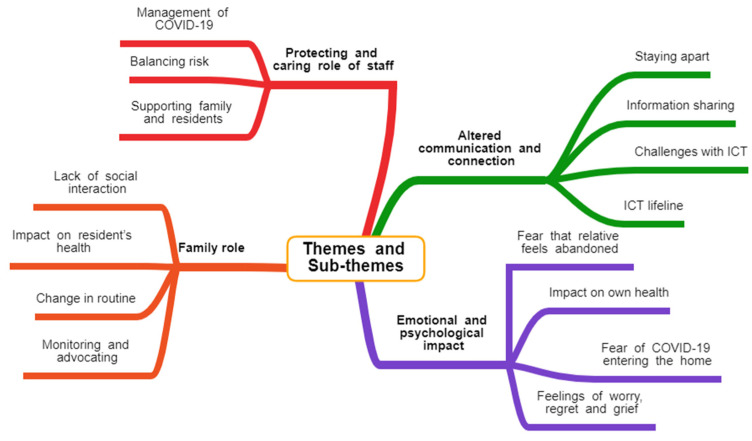
Themes and subthemes identified from a thematic analysis of the shared experiences of the 120 caregivers of people living in LTRCFs in Ireland.

According to reflective accounts of the caregivers who responded, the COVID-19 pandemic had a significant physical and emotional impact on both residents living in LTRCFs in Ireland and their relatives and friends. The loss of regularly meaningful physical contact with family was gravely felt by nearly all respondents and the sudden withdrawal of visitors potentially caused harm to both visitors and residents. Following the analysis of reflective accounts, four main themes and fifteen subthemes were constructed as summarized in Figure 1. The main themes are altered communication and connection; emotional and psychological impact; protecting and caring role of staff; and family role. 

### 3.1. Altered Communication and Connection

This theme relates directly to the changes faced by families with the rapid pivot from in-person to remote engagement during the first 3 months of ‘lock down’ and illuminates some of the main barriers and opportunities presented by information and communication technology (ICT) solutions. Within this theme, four subthemes were identified: staying apart; information sharing challenges with ICT and ICT lifeline.

#### 3.1.1. Staying Apart

With family members unable to visit, and engage in care, key aspects of caregiving, for both the person and the family member, were interrupted. As full restrictions began to ease families reflected on the notion of still staying apart, separated by Perspex screens and masks. Window and garden visits, while generally a welcome breakthrough was described by some as further impeding that personal connection. For example, one relative stated

*“Now that the restrictions are being eased a little, and there is talk of visits, I’m not sure it will be that great: I’m assuming I won’t be able to hug her or hold her hand, I’ll have to wear a mask and she won’t be able to see me smile at her, so I think it will be quite hard to communicate, given that she has such problems with her speech: sometimes a smile has been the most meaningful and reassuring expression of love between us. But I am so very grateful that she is still here...”*

#### 3.1.2. Information Sharing

Information sharing was described as difficult and with mixed reactions. Some felt that regular phone calls with updates to family were great to keep one informed, however, others felt that the same information was given to all families and was generically delivered rather than relating to the specific care needs of their relative.

*“It was very frustrating getting information, I know the Nursing Home had a lot of staff that went down with COVID-19, I think Government should have sent (an) Army of hired Healthcare in. There were weeks we couldn’t talk to anyone as there were no staff. They sent us a text that was sent to everyone. It was very upsetting…”*

Owing to issues, such as high staff turnover and the busy care setting, there were reported challenges in making contact with loved ones. The contact described was led by family, informally.

*“It is so distressing worrying about him now as it is all new staff and patients for him now to contend with- how unfair is that? We can’t see the room he is in. We ring every day since COVID-19 to see how Dad is doing and I don’t know if we didn’t ring would we ever have gotten a phone call only to say he was being moved. I don’t sleep at night now the worry and stress has affected my life and my family”*

Respondents also mentioned barriers to communicating with staff, where English was not their first language, hindering information exchange.

*“Most of the staff in her facility are foreign with limited English and it is difficult to communicate exactly what my mother needs…”*

#### 3.1.3. Challenges with ICT

Some respondents noted that advance care planning conversations were absent, particularly if their relative contracted COVID-19. Some families felt that phone calls were rushed, and that staff were unable to deal with the volume of daily phone calls in the initial weeks of the pandemic. Other challenges that emerged related to the use of video calls, which some families described as causing distress and confusion for their relatives with dementia. One person suggested that there should have been a dedicated ‘tech person’ employed to support this shift to communicating through applications on smartphones and tables.

*“My mother is blind as well as suffering from dementia so the use of digital devices while offered by the care home was not of any value. Even phone call proved to be useless she refused to talk! So I sent cards and letters which staff read to her.”*

It is clear from the first quote that a diagnosis of dementia and other communication impairments hinder synchronous communication, nonetheless more traditional forms of communication appear to have been encouraged and supported by staff. One national initiative by the Irish postal services was ‘free postage to care homes’, which was aimed at keeping communities connected. 

#### 3.1.4. ICT Lifeline

Conversely ICT was described as a comfort and one person described that although their relative doesn’t know who they are on the calls the staff know that it’s important for them to see their loved one on a regular basis through face time. Others stated that their relative responded really positively to using video calls and hailed them as a lifeline.

### 3.2. Emotional and Psychological Impact

This theme describes the emotional and psychological impact experienced by families. It was evident throughout the responses that families were stressed, angry and frustrated. Much of the frustration was related to a lack of clear guidance on lines of commination and uncertainty surrounding the timeline for relaxing restrictions. Fear appeared to dominate the emotions of many, this is reflected in the four subthemes that emerged: fear that a relative feels abandoned; impact on own health; fear of COVID-19 entering the home; and feelings of worry, regret and grief.

#### 3.2.1. Fear That Relative Feels Abandoned

The rapid onset of the visiting restrictions meant that many family members had to stop visiting and a fear that their relatives would not understand why they had suddenly stopped calling to see them was very evident. This was associated with a feeling of guilt. One daughter explained that:

*“As a daughter I have worried about my mother. I was afraid that I would never see her again and that she would die alone and not know why her family did not come to her”*

#### 3.2.2. Impact on Own Health

Respondents discussed how the visiting restrictions had impacted their mental health. Nine families used the word heartbroken to describe how they felt. Others stated that they felt depressedand had commenced on medication to cope with the situation.

*“I have found the restrictions very distressing…. I have found the past few months so hard and draining that I have had to go on anti-depressants.”*

#### 3.2.3. Fear of COVID-19 Entering the Home

Another prevailing concern was fear of COVID-19- entering the nursing home and this was both from the perspective of the family bringing it in when restrictions are relaxed and just general anxieties that there would be an outbreak and they couldn’t do anything to help. This appeared to result in feelings of loss of control, being described by many respondents as “helplessness”.

#### 3.2.4. Feelings of Worry, Regret and Grief

Within the responses, there were varied accounts of the emotional impacts of not being able to see family members, from helplessness to isolation and worry. Some expressed regret and guilt for not looking after their relative in their own home and many were grieving for lost time and a lost connection with their loved this was particularly prominent in those with relatives with dementia. One person described that the bond with their relative was fading as each day passed.

*“This has affected me and my family hugely. We feel that we are on the outside looking in. We are not included in any of the care at all due to COVID-19 restrictions and this makes us feel very helpless.”*

### 3.3. Protecting and Caring Role of Staff

This theme discusses family perspectives of healthcare staff during COVID-19, described across three subthemes: management of COVID-19; balancing risk; and supporting family and residents.

#### 3.3.1. Management of COVID-19

Despite the clear toll that the restrictions were having on family members’ health and well-being, the protecting and caring role of staff within the homes and their trojan efforts to keep everyone on staff was lauded by the majority of respondents.

*“The staff for the most part have been fantastic, and we appreciate the sacrifices which they have made and continue to make to safeguard the health and well-being of the residents. I will be forever grateful to them for their care and support to my lovely dad”*

Despite some feeling that nursing homes were not well supported by the (Irish) government, particularly in the early days of the pandemic, some families felt that senior management within the homes fought hard to prepare staff and address resource issues. Furthermore, the management of COVID-19 outbreaks was mentioned in the context of staff being professional and adhering strictly to guidelines, which provided reassurance to families. Some saw the absence of COVID-19 cases in their relative’s nursing home as a sign of quality care and praised staff for their diligence and perseverance in keeping it out.

*“Thankfully there were no cases in the Nursing Home and I believe that was down to the Management who didn’t take a day off for three months to keep their Residents and Staff safe. They communicated with us at least twice a week and also made time to contact us a few times a week with videos, texts and videocalls with Mum. We can never thank them enough. They were well prepared for the pandemic and kept on top of all the guidance. They informed us of every change made by the Dept of Health before it happened and we were happy to receive plenty of nursing and medical progress information on our Mother also.”*

#### 3.3.2. Balancing Risk

Owing to concern regarding the impact on day-to-day life, respondents called for a review of the current restrictions, which prevent interaction or engagement outside of the LTRCF, and consideration of the risk-quality of life continuum.

*“…Quality of her life must be balanced against the risk of infection. This cannot go on for ever, no touch, no sitting in the garden, no outings, no visits to my house for a meal or just a change of scene”.*

Perhaps owing to the emergent/evolving nature of COVID-19, there was a sense that only universal restrictions were applied, a reluctance to explore alternative options or consider the needs of different residents.

*“The refusal to acknowledge other options for dealing with this outbreak of viral illnesses and the mishandling of how best to treat and care for the very elderly, vulnerable and already sick people at home or in Nursing/care homes has shown up the lack of skill/management in preventing some deaths and containment of spread of infection.”*

This was particularly the case in LTRCFs for persons living with intellectual disability, where a blanket approach to visitor restriction was deemed overly severe.

*“My son is neither ’over 70 nor medically vulnerable’ but the same visitor restrictions apply - not really in line with disability policy…”*

While many found the restrictions very difficult, they reflected on the necessity to keep the virus out and there was a sense of acceptance on balance with some describing it as:

*“a price they were willing to pay”* to keep their relative safe.

#### 3.3.3. Supporting Family and Residents

It was evident from the respondents’ reflections that many felt supported by staff and that an effort was being made to maintain a family-resident centered care approach to care, despite the obvious barriers.

*“It is a tough time for older people, but the reality is she is very well cared for both physically and emotionally by wonderful staff and she is safe and protected.”*

Confidence in staff was evident and some of the adjectives used to describe staff were *wonderful, supportive, professionals, kind, patient*. Indeed, many stated that they would be “*eternally grateful*”.

### 3.4. Family Role

The final theme relates to the changing role of the family and is discussed across four subthemes: lack of social interactions; impact on resident’s health; change in routine; and monitoring and advocating. One respondent eloquently captures the essence of the huge impact that the visitor restrictions have had on the lives of so many families and how they perceived that the absence of their role has impacted negatively on their relative’s well-being.

*“The home has been brilliant. However, lack of visiting, social activities and usual exercise has meant my Father’s health has declined a lot faster. He no longer remembers who I am which is extremely hard, but the staff are very aware and caring to both of us. His mobility has reduced which has affected his balance and initially at the beginning of COVID-19 he felt frustrated, confused and lonely for family. I feel that if I could have continued to see him on a regular basis, he would still know me. The staff tried phone calls and WhatsApp videos, but he didn’t like either of them or got frustrated. I totally understood why the restrictions were in place and I’m thankful that he is healthy and happy but I’m sad to have lost our connection”*

#### 3.4.1. Lack of Social Interaction

There was a sense that the lack of daily activities, and the lack of stimulation and interaction with others, impacted the mental health of residents.

*“… I note that (his) psychological state has deteriorated. I don’t know how much stimulation he is receiving. Prior to COVID-19, he was used to daily one-to-one conversation. Now he makes no effort to communicate through a Perspex screen.”*

Visiting was described as an activity that nurtured memories, reduced loneliness and isolation and was essential to their relative physical and mental health.

#### 3.4.2. Impact on Resident’s Health

Respondents described changes to their family member’s daily functioning. They observed what they believed to be deterioration in activities of daily living.

*“Not an hour goes by that I think of him and worry is he ok, is he being cared for? I miss taking care of him and talking to him, he is my family…Before he was hospitalized he could feed himself and walk, now he has to be fed, can’t walk and (is) incontinent.”*

Respondents discussed changes they had observed in their family members, for example, a perceived negative impact on cognition and general health status.

*“My mother has slipped further into her dementia. She was used to visitors 4 times a week and then dropped to none. Her level of consciousness has decreased as has her speech.”*

Beyond the input into physical care needs, families noted that sometimes relatives just need to be in the presence of family to support positive well-being and connection with the past.

#### 3.4.3. Change in Routine

Several respondents expressed concern about the disruption to the normal routine, and a lack of daily activities in LTRCFs, as a result of changes associated with COVID-19.

*“Residents have been deprived of family visits and the ability to take part in important activities such as music, bingo, art and physical activities which are so important to their mental and physical health.”*

The discontinuation of daily events, such as shared mealtimes meant that for many people, significant isolation within LTRCFs was a concern.

*“Our worlds turned upside down on 7 March. At end-of-life, my mum is now 90, the future looks bleak. Daily mass, communal meals, daily activities, hairdresser, visits out every Friday and Sunday to see children and grandchildren in their homes all stopped. She is confined to her bedroom. Like a prison room with nothing to look forward to. Care with (activities of daily living) continue but there is nothing else.”*

It was felt that the many changes to routine, activities and reduced contact with family had led to a global decline in their loved ones.

*“The home has been brilliant. However, lack of visiting, social activities and usual exercise has meant my Father’s health has declined a lot faster. He no longer remembers who I am which is extremely hard … His mobility has reduced which has affected his balance and initially at the beginning of COVID-19 he felt frustrated, confused and lonely for family…If I could have continued to see him on a regular basis, he would still know me.”*

#### 3.4.4. Monitoring and Advocating

It was felt that owing to the inability to visit, family members were less able to monitor the care the person received in the LTRCF. In the past, many reported having supplemented extra care, as and when necessary.

*“It is very difficult not being able to visit for both the patient and me. If anything was out of place you would have it attended to calling in. Now you feel helpless when you hear of something amiss.”*

The visiting restrictions led to a reported reduction in instrumental activities of daily living, particularly when families had typically assisted with these.

*”I can’t go inside the (nursing home) door. I can’t fix her radio. I can’t sort out her mobile. She is so confused I don’t know what is true/real in her experience. I can’t see for myself. The staff are kind but she is difficult and they struggle to communicate with her. I can’t act as intermediary.”*

While keen to continue monitoring care from afar, there were limited means to do this. There was particular concern when their family member had a diagnosis of dementia or similar, and where the family had previously acted as advocates for the person.

*“The first few weeks were so distressing for me, even though I knew that the staff are kind and take good care of her. I was worried that they would be so busy and preoccupied that they wouldn’t have time to spend on her… Mum is not a loud, demanding or complaining person, so I feared she might be lost without me to speak up for her, notice little things that needed attention and do certain things for her.”*

## 4. Discussion

This study examines the perceived experiences, concerns and impacts of families and friends of residents in LTRCFs during the early stages of the COVID-19 pandemic in Ireland. The themes identified show that a blanket approach or one-size-fits-all approach to visitor restrictions can be harmful to both residents and their close friends and relatives. These themes also highlight important lessons that can be learned from the first wave of the pandemic and future such events.

### 4.1. Role of Family in Care Provision

Family involvement in nursing home care is a multidimensional construct that entails visiting, socioemotional care, advocacy, and the provision of personal care. Its importance should not be underestimated. A sense of meaning and worthiness can be derived from connectedness for families [24]. Continued family relationships often have significant meaning to family members, when this is disrupted family carers experience a loss of self-identity, loss of closeness, and a loss of purpose [11]. The findings from this study provide us with insight into the experience and impact of family’s absence in the provision of their relative’s care during the first wave of the pandemic in Ireland. The reflective accounts illustrate the many negative effects that visiting restrictions have had, from disrupted communication and loss of meaningful connection between families to considerable impacts on emotional and mental well-being. Findings demonstrated the significant protecting and caring role played by staff in LTRCFs and underscored the crucial, multi-faceted role played by families of persons living in LTRCFs.

There has been a significant underestimation and undervaluing of the caregiving role of family and the visitors’ restrictions havehighlighted the need to recognize how families are essential care partners who provide physical, psychological and emotional support [25]. Canada has been leading in this regard and has developed guidance on finding the right balance and supporting family caregivers as partners in care [26], making the distinction between those who frequently provide essential personal care to residents and those who visit for social reasons, occasionally. Moreover, while the role of ‘general visitors’ in providing company and social engagement should not be underestimated, care provision provided and informed, usually by immediate family, should be given priority as their reduced input may impact negatively on residents’ care. This distinction was affirmed by a Delphi panel of 21 experts from Canada and the United States [27]. Further to this Low et al. [28] (p. 2) have recommended that “Family caregivers’ should be designated as essential partners in a resident’s care during the pandemic and be able to have more frequent, longer hands-on visits if they can be supported to do so safely and that Care homes should receive additional government funding and support to implement safe visiting practices” [28]. Many felt that the lack of social interaction and change in routine led to cognitive and physical deterioration of their relative (including worsening of mood and increase in behavioral problems) [29,30]. It was clear given the frequency and purpose of visits of the respondents that the absence of their input in care provision and social activities would leave a visible impact. There was also a sense of fear of not knowing how their relative was managing on a day-to-day basis and whether staff would miss subtle changes or perhaps not understand their relatives’ needs without the family being there to advocate, monitor and contribute to care planning decisions. Most wanted to be more involved and missed their caring role which provided them with a sense of meaning and purpose.

### 4.2. Impact on Family, Residents and Staff

The study findings add to the evolving body of knowledge on the direct and indirect implications of COVID-19 and the attendant public health measures. Findings indicate a clear need for the provision of ICT solutions that will meaningfully support remote engagement in care. Effective communication is the cornerstone of quality care, it improves patient outcomes, provides emotional support to families, and informs good decision-making processes [31] but without traditional lines of communication, these aspects of care were threatened and staff, family and residents alike were required to rapidly adopt communication solutions to maintain connections and continue to inform care [32]. Quality communication provides emotional support to families, builds trusting relationships and informs good decision-making processes [33,34]. However, the COVID-19 pandemic derailed resident-family centered care, impacting face-to-face communication processes and procedures between family and care facilities. Some families have reported that prior to the pandemic they had several daily visits to the LTRCF, to support the care of their loved one who was confused, distressed, or in pain. Noting they were uniquely positioned to care for their relative with intimate knowledge of their life story.

Findings highlighted how the restrictions have impacted the person in care, with incidences of adversely impacted health, functioning, cognition and mental health [18]. Responses from 70 family carers of people with dementia in Canada expressed very similar concerns with over half (51%) reporting an increase in dementia-associated responsive behaviors since the start of the pandemic [35]. Similar concerns were raised in Finland amongst 41 interviewed carers who expressed concern for their relative’s decline accompanied by anxiety, grief and severe stress [36]. Frustration with not being able to touch their relatives was also expressed [36].

“Group activities in nursing homes were also prohibited. As a consequence, the residents of nursing homes became more socially isolated” [37]. Emphasis should be on physical distancing, rather than social distancing, and with the right types of support from community organizations, friends, family, and/or neighbours many older adults can successfully adapt to post pandemic living [38] People living with dementia who rely on in-person support (rather than information technology) may experience loneliness and may become withdrawn [37].

The impact extends to the family members, who report adverse mental and physical health, in addition to a perceived decline in relatives living in care, as a result of the significant changes. Many of the family members expressed high levels of concern for their relatives and an emphasis was placed on the fact that they could not take care of usual activities or advocate for their needs, particularly for those with dementia. These reports are consistent with a study of 958 family caregivers of people with dementia in the Netherlands which found higher levels of worry amongst more active carers [39]. Similarly, this Irish cohort suggests that carers of those with cognitive impairment have lower wellbeing and tend to visit more frequently [15]. Further, in addition to worry, family carers may themselves experience social isolation or loneliness while not being able to visit their relative [35,39].

In addition to safeguarding measures for virus infection, Wang et al. (2020) have highlighted the need for mental health and psychosocial support for staff working in long term care settings [37]. Our findings indicate a need for this support to be extended to family caregivers of persons living in LTRCFs.

### 4.3. Model of Care Delivery

Consideration of the prevailing model of nursing homes and LTRCFs is warranted, including the environmental structure and social environment. A future policy shift should include key aspects of family support while drawing a distinction between family caregivers and visitors and effectively balance the social and emotional value of visits against the risks of infection and polypharmacy [18,27,28,40,41,42,43]. This international literature does not support blanket bans on visitation and the removal of residents’ right to face-to-face meetings should be a last resort. Individual cases can be complex and ethically challenging [44], and residents and their families should receive regular consultation during the planning process [27,28,41,42].

The model of LTRC care in Ireland has been questioned—both in design and delivery [45]. There are emerging opinions internationally regarding improved layout to support improved infection prevention and control measures and integration of safe visiting practices [28]. Resources are recommended for capital and environment planning and a model of care with a shift in design to smaller household models of residential care resembling a family home (accommodating 6–12 people) [45]. Construction of small-scale shared housing facilities, such as these is common in some European countries, such as Germany, Sweden and the Netherlands [46,47]. This shift to creating community-based environments will require government support and long-term policy and resource investment [45].

### 4.4. Limitations and Strengths

This study has several limitations. It was conducted at a single time-point and may not be representative of the experiences throughout subsequent waves of the pandemic. This being said, the study was conducted during the initial intense ‘lock down’ of nursing homes in Ireland in 2020 when the greatest potential for impact could be expected. Subsequent waves of the pandemic brought about learning and such draconian restrictions have not been perpetuated. This study highlights the potential and unforeseen consequences of the initial ‘lock down’ which represents an important source of learning for subsequent public health restrictions and future pandemics. Another limitation relates to how data were collected, in that, the qualitative element of the survey comprised open-ended questions and comments as part of a larger cross-sectional web-based survey. Given the restrictions, during the first wave of the pandemic, this method acted as a means of casting a wider net, to help identify issues not covered by the closed questions in the questionnaire. Providing this additional opportunity for further information allowed participants to provide more details about issues and concerns they experienced. Also, the open-ended questions reduced the constraints imposed by closed ended questions. Given the large number of responses and lengthy narratives provided, there was an abundance of context and rich accounts offered, hence it was necessary to analyze the data and convey the consequences of these restrictions for individuals living in LTRCF from the perspective of their families during the first wave of the COVID-19 pandemic.. Although this study does not communicate the longitudinal impact of the visitor restrictions, it does serve to provide valuable insight into the impact of the blanket visitor ban which is now recognized as a last resort but still practiced in certain countries adopting a ‘zero-COVID’ policy.

## 5. Conclusions

The COVID-19 pandemic has brought with it many challenges, none more so than how best to manage the care of the most vulnerable and frailest in society, residents in LTRCFs. This research highlights that taking a blanket approach to visitor restrictions with a failure to distinguish between those who provide essential emotional connection and often personal care to residents and those who visit for social reasons, has the potential to cause harm to both visitors and residents. We suggest that a partnership approach to care provision is required as this pandemic continues and as a template for future similar events. This should encompass strategies that balance safety concerns with the psychosocial needs and emotional well-being of families and their relatives during times of self-isolating or restrictive measures. Tailored, individualized approaches that adhere to protective public health measures rather than uniform restrictions will likely strike the best balance, though research is needed to develop and operationalize such policies.

## Data Availability

The qualitative date has not been made available through open source.

## Supplementary Materials

The following supporting information can be downloaded at: https://www.mdpi.com/article/10.3390/ijerph19116559/s1, Table S1: Descriptive characteristics of the 120 survey participants that provided feedback on their experiences and were included in this study compared with those who did not provide feedback and were excluded from the study.
