# Supplementary material for: The Experience of COVID-19 Visitor Restrictions among Families of People Living in Long-Term Residential Care Facilities during the First Wave of the Pandemic in Ireland
Source: Int J Environ Res Public Health. 2022 May 27;19(11):6559. doi: 10.3390/ijerph19116559 (PMC9180630; doi:10.3390/ijerph19116559)
Supplement: Supplementary file 1 [file ijerph-19-06559-s001.zip › ijerph-1660833-supplementary.pdf]

International Journal of *Environmental Research and Public Health*

MDPI

Table S1. Descriptive characteristics of the 120 survey participants that provided feedback on their experiences and were included in this study compared with those who did not provide feedback and were excluded from the study.

	Survey participants with feedback (included) N = 120		Survey participants without feedback (excluded) N = 105		P-value** for the difference
	Number	Percent	Number	Percent	
**Demographics (reported by respondents)**					
Age					0.485
18-44 years	19	16%	24	23%	
45-54 years	47	39%	41	39%	
55-64 years	36	30%	29	28%	
65+ years	18	15%	11	10%	
Female sex	99	83%	85	81%	0.764
Family of LTRCF resident	114	95%	91	87%	0.028
Employed	77	64%	76	72%	0.188
Lives alone	29	24%	14	13%	0.039
**Resident characteristics (reported by respondents)**					
Institutionalized for at least a year	88	73%	73	70%	0.527
Resident with severe dementia	41/109**	38%	32/93**	34%	0.636
Resident receiving end-of-life care	13/118**	11%	9	9%	0.541
**Location of LTRCF (reported by respondents)**					
Geographic area					0.406
Northwest (Connacht/Ulster)	16	13%	18	17%	
East (Leinster)	56	47%	40	38%	
South (Munster)	48	40%	47	45%	
Urban area	51	43%	37	35%	0.265
**Caregiver role (reported by respondents)**					
Usual frequency of visits					0.462
At least twice a week	76	63%	60	57%	
Weekly to fortnightly	38	32%	36	34%	
Several time a year or less	6	5%	9	9%	
Provide care (always/usually do)	28	23%	20	19%	0.434
Do activities with resident (always/usually do)	64	53%	49	47%	0.318
**Impact of COVID-19 on visitor and resident (perceived/reported by respondents)**					
Resident tested positive for COVID-19	15/119**	13%	6	6%	0.077
Impacted communication with staff significantly	49	41%	32	30%	0.106
Decreased satisfaction with care	38	32%	21	20%	0.047
**Perceived impact of visitor restrictions (on LTRCF resident)**					
Resident coping well					0.059
Yes	31	26%	42	40%	
Don't know	22	18%	19	18%	
No	67	56%	44	42%	
Change in mood (yes)	74	62%	50	48%	0.033
Change in functioning (yes)	61	51%	37	35%	0.024
Change in memory (yes)	65	54%	49	47%	0.247

LTRCF: Long term residential care facility; *Pearson Chi Square test; **These variables have missing responses. All other variables had full data.
